# Secondary Immunodeficiency

**DOI:** 10.1186/s13223-024-00925-4

**Published:** 2025-01-27

**Authors:** Persia Pourshahnazari, Stephen D. Betschel, Vy. H. D. Kim, Susan Waserman, Rongbo Zhu, Harold Kim

**Affiliations:** 1 Division of Allergy and Immunology, Department of Medicine, University of British Columbia, Vancouver, BC Canada; 2 Division of Allergy and Immunology, Department of Medicine, St. Michael’s Hospital, University of Toronto, Toronto Ontario, Canada; 3 Division of Immunology and Allergy, Hospital for Sick Children, Toronto, ON Canada; 4 Department of Paediatrics, University of Toronto, Toronto, ON Canada; 5 Division of Clinical Immunology and Allergy, Department of Medicine, McMaster University, Hamilton, ON Canada; 6 Division of Clinical Immunology and Allergy, Department of Medicine, Western University, London, ON Canada

## Abstract

The field of medicine is constantly changing and, as healthcare providers, we are fortunate to be practicing in a time when patients are living longer and novel therapeutic options continue to evolve. However, these new advances may be associated with adverse effects that practitioners need to be aware of. Some of these impair the immune system leading to secondary immunodeficiencies (SID) that increase host susceptibility to infections and other complications. The causes and consequences of these SID are extremely broad, and a detailed review is beyond the scope of this article. The goal of this primer is to provide a general overview and understanding of common conditions and therapies leading to SID, as well as a guide to the assessment and management of patients with SID.

## Key take-home messages

- SID occur when factors extrinsic to the host immune system lead to impaired immune responses.
- Prompt recognition and appropriate management of SID are important to reduce patient morbidity and mortality.
- Patients affected by SID are highly heterogeneous and warrant a multidisciplinary approach to help optimize care.

## Background

**Fig. 1:**
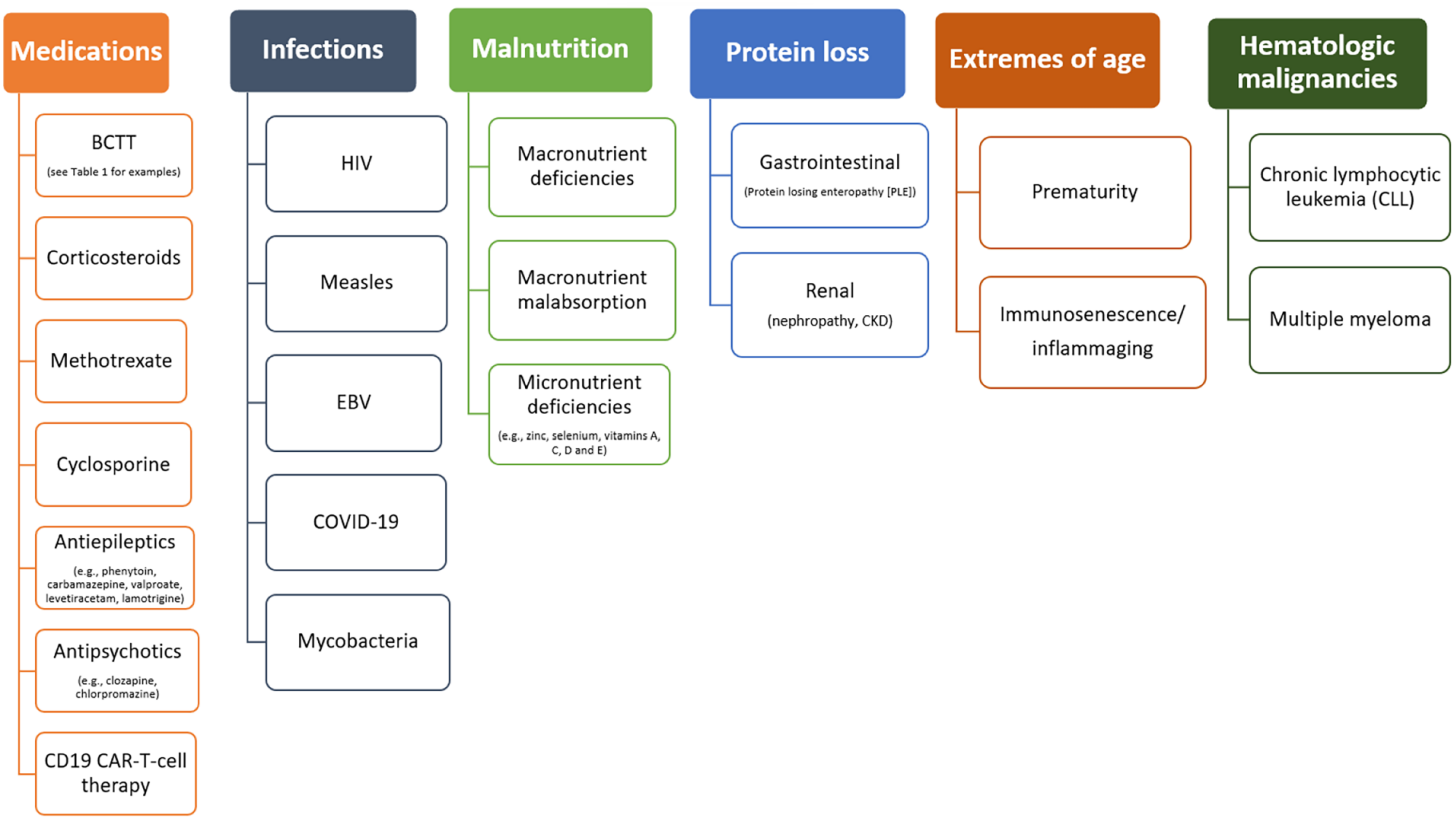
Possible causes of SID*. *BCTT* B-cell targeted therapy, *CAR-T-cell* chimeric antigen receptor T-cell; *CKD* chronic kidney disease, *COVID* coronavirus, *EBV* Epstein–Barr virus, *HIV* human immunodeficiency virus, *SID* secondary immunodeficiencies. *Not an exhaustive list of potential causes of SID

Secondary immunodeficiencies (SID) are due to factors that are unrelated to inborn errors of immunity (IEI) (also referred to as primary immunodeficiency disorders [PID]) (see *Inborn Errors of Immunity* article in this supplement). There are several causes of SID (Fig. 1), and they can affect both innate and adaptive immune responses (see *Introduction to Immunology & Immune Disorders* article in this supplement) and/or multiple arms of the immune system simultaneously. This heterogeneity can make both evaluation and management challenging [1, 2]. SID can be transient and reversible in some cases, but in others, impairments are persistent.

In adults, SID are more common than IEI, and the causes of SID are steadily increasing [1, 2]. Factors such as population growth, demographic shifts (i.e., older/aging populations), increasing prevalence of certain malignancies, and use of novel immunosuppressive agents (such as biologics and chimeric antigen receptor [CAR] T-cell therapy) are contributing, in part, to this trend [1, 2]. Between 2005 and 2015, for example, there was a 26% increase in the incidence of chronic lymphocytic leukemia (CLL)—one of the malignancies commonly associated with SID [3]. Approximately 80% of patients with CLL will experience infectious complications related to SID, and the primary cause of mortality in these patients remains infection [4]. It is important to recognize and treat SID promptly to avoid complications that can lead to morbidity and mortality. In this primer, we provide a general overview of several conditions and treatments that cause SID and the assessment and management of patients with these immunodeficiencies. It should be used as a framework for understanding this topic, and it is best utilized in conjunction with more detailed published reviews of SID (see Tuano et al. 2021;127(6):617–626 and Otani et al. J Allergy Clin Immunol. 2022;149(5):1525–1560).

### General considerations

SID encompass a highly heterogeneous group of conditions caused by a variety of factors, such as malnutrition, infections, medications, malignancies and autoimmune diseases (Fig. 1 provides an illustrative, though not exhaustive, summary of some possible causes of SID) [2]. As a result of their heterogeneity, patient populations with SID can vary significantly in terms of disease entities, natural history, and long-term prognosis. Furthermore, there is a paucity of high-quality data available to inform clinical practice regarding the diagnosis and management of SID. The limited number of randomized controlled trials (RCTs) have used variable definitions, study protocols, outcomes, and supportive care measures, making it difficult to translate research findings to clinical practice [2]. As a result, most published recommendations for the diagnosis and management of SID are based on expert opinion or clinical experience and/or are extrapolated from data generated by older SID studies or studies of IEI [2, 5, 6].

### Specific causes of SID

#### Medications

Many different medications can cause SID (see Fig. 1), and the mechanisms by which they do so are highly variable [1, 2]. Immunosuppressive medications are often prescribed in combination and can lead to additive immune impairments and increased infectious risks. Prescribers of medications with high risk of causing SID should evaluate immunoglobulin (Ig) levels prior to medication initiation. An overview of some of the key medication causes of SID is provided below.

##### B-cell targeted therapies (BCTT)

Secondary hypogammaglobulinemia (SHG) (a humoral SID characterized by reduced Ig levels due to medications or disease processes that lead to decreased antibody production or increased antibody loss) commonly occurs after BCTT, which is used to treat a wide array of conditions including hematologic malignancies and autoimmune conditions (see Table 1). Although most of the literature regarding BCTT-associated SHG and its infectious complications is on rituximab (RTX), a chimeric anti-CD20 monoclonal antibody [1, 2, 7], other BCTT have also been associated with SID (see Table 1) [1, 7–18].

**Table 1:**
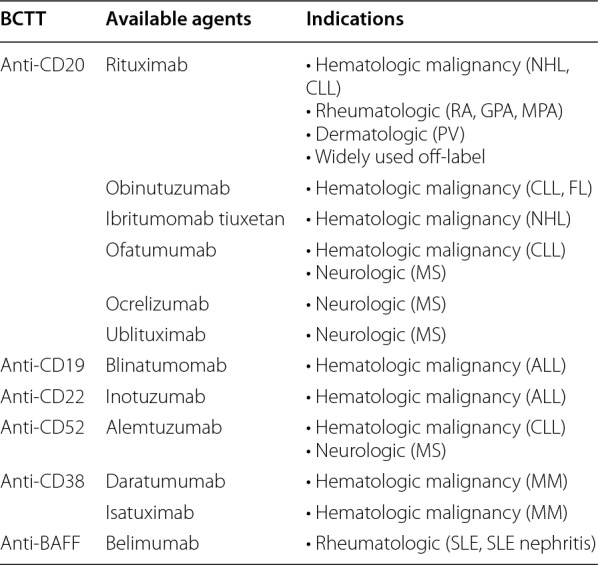
BCTT associated with SID and their indications

*BCTT* B-cell targeted therapy, *NHL* non-Hodgkin lymphoma, *CLL* chronic lymphocytic leukemia, *RA* rheumatoid arthritis, *GPA* granulomatosis with polyangiitis, *MPA* microscopic polyangiitis, *PV* pemphigus vulgaris, *FL* follicular lymphoma, *MS* multiple sclerosis, *ALL* acute lymphoblastic leukemia, MM: multiple myeloma, *SLE* systemic lupus erythematosus

The immune effects of RTX include B-cell depletion, neutropenia and SHG, which can increase the risk of infections. Although rates of SHG with RTX vary depending on the underlying condition and other immunosuppressive treatments being used, B-cell depletion and SHG can persist for years after discontinuation of the drug. Prior to starting RTX, immunoglobulin levels should be checked to identify any pre-existing hypogammaglobulinemia (HG). One potential benefit of this approach is to identify patients with underlying immune dysregulation due to IEI, as these patients are at higher risk of developing infectious complications [1, 2]. For example, common variable immunodeficiency (CVID) is one of the most common IEI and is known to predispose patients to B-cell lymphomas which are commonly treated with RTX and other BCTT.

##### Corticosteroids

Corticosteroids have broad effects on gene transcription that can lead to anergy and apoptosis of lymphocytes [1, 2]. The immunologic effects of long-term and/or high-dose systemic corticosteroid use include CD4 T-cell lymphopenia and SHG, leading to an increased risk of infection. Corticosteroid use has a greater effect on IgG levels than IgA and IgM. Even short courses of systemic corticosteroids have been associated with a transient drop in serum IgG, which can last for several weeks after stopping the corticosteroid.

Although a wide variety of viral, bacterial, and fungal infections have been observed with prolonged use of corticosteroids, increased susceptibility to these infections is largely due to corticosteroid-induced lymphopenia rather than to SHG.

##### Antiepileptics

Antiepileptics, such as phenytoin, carbamazepine, valproate, levetiracetam, and lamotrigine, can cause SHG, and some (phenytoin, carbamazepine) also cause lymphopenia [2]. The infectious risk from SHG associated with use of these medications is not yet well-defined.

##### CD19-targeted chimeric antigen receptor T-cell (CAR-T-cell) therapy

CD19-targeted CAR-T-cell therapy is a novel therapeutic that uses engineered T-cells with chimeric antigen receptors against CD19 for the treatment of acute lymphoblastic leukemia (ALL) and adult B-cell lymphomas. Although it is a highly effective therapy for these disorders, B-cell aplasia and SHG are frequent and often prolonged complications of CAR-T-cell therapy [1, 2].

#### Infections

Certain pathogens can cause both temporary and persistent immune changes leading to impaired host immune responses. Below we describe a few of the most important pathogens that can cause SID (Table 1).

##### Human immunodeficiency virus (HIV)

HIV infection is a well-known cause of SID, which is referred to as acquired immunodeficiency syndrome (AIDS). HIV infects CD4 + T-cells, as well as other cells expressing CD4 [1, 19]. Infection leads to depletion in CD4 + T-cells and the creation of large viral reservoirs within the body, resulting in an increased risk for infection. This is particularly true of opportunistic infections and reactivation of latent pathogens. HIV also accelerates immunosenescence (the decrease in immunologic competence that occurs with age) and inflammageing (the low-grade, chronic inflammation that develops with age [19].

##### Measles

Measles virus infection has both short- and long-term effects on immunity. T- and B-cells are directly infected during the acute phase, inducing transient lymphopenia and impairment in lymphocyte function [1, 20]. Over time, memory B-cells and plasma cells are reduced, leading to an increased risk of infection. [20]. In fact, the increased susceptibility to other infections in measles survivors continues for years after apparent recovery from the virus.

##### Coronavirus (COVID)-19

Severe acute respiratory syndrome coronavirus 2 (SARS-CoV-2) emerged as a global pandemic in late 2019. Acute infections with SARS-CoV-2 can cause significant lymphopenia and lymphocyte dysfunction, which correlate with disease severity [21]. Stimulation of secretion of large amounts of cytokines contributes to uncontrolled immune dysregulation and cytokine release syndrome [22, 23]. Studies examining the long-term effects of SARS-CoV-2 on the immune system have shown prolonged immune dysregulation in a subset of individuals, which may be implicated in “long COVID” [24]. However, the long-term clinical effects are currently unknown.

##### Mycobacterium tuberculosis

*M. tuberculosis*, the causative agent of tuberculosis, is an intracellular pathogen that is well adapted to establish infection and evade immune clearance, making it one of the leading causes of mortality by a single infectious agent worldwide [1, 25]. The pathogen inhibits the activation of macrophages, accelerates apoptosis of antigen-presenting cells (APCs), and impairs lymphoproliferative responses, leading to chronic inflammation and an increased risk of secondary infections in chronically inflamed tissues.

#### Malnutrition

Malnutrition is the leading cause of SID worldwide. The impact of malnutrition on the immune system is multifaceted. Macronutrient deficiencies and malabsorption can affect all arms of the immune response, and micronutrient deficiencies can have specific negative effects on the development and maintenance of the immune system. Zinc deficiency, for example, is associated with lymphopenia, reduced Th1 cytokines, and impaired mucosal immunity [1]. Vitamin A deficiency is associated with impaired epithelial barrier function and reduced CD4 and CD8 T-cell numbers.

#### Protein loss

Protein loss, which can occur through the gastrointestinal (GI) tract, kidneys, and skin (e.g., vis-à-vis burns or skin trauma), can affect both humoral and cellular immunity. Uremia in chronic kidney disease (CKD) can also affect innate immune cells.

##### GI protein loss

Protein-losing enteropathy (PLE) occurs secondary to the loss of protein in the GI tract from GI disorders [1, 2]. SHG can occur in PLE when net enteral protein loss exceeds the body’s ability to synthesize sufficient protein to replace what was lost. PLE is associated with an increased risk of infections from SHG, lymphopenia, and malnutrition (i.e., loss of macronutrients and fat-soluble vitamins) [2].

##### Renal protein loss

In nephropathy, urinary protein loss can lead to SHG and increased risk of infections [1, 2]. This can be complicated by the fact that many commonly used medications to treat nephrotic syndrome, such as BCTT and corticosteroids, can also induce SID. In CKD, immunodeficiency is due to proteinuria/protein loss as well as uremia, which affects both the innate and adaptive arms of the immune system.

#### Extremes of age

##### Prematurity

Both innate and adaptive mechanisms are impaired in premature infants. Impairments to innate immunity include reduced Toll-like receptor responses with decreased production of proinflammatory cytokines, impaired neutrophil function and tissue migration due to reduced cell surface adhesion molecules, as well as decreased major components of the complement cascade [1, 26]. Transplacental transfer of maternal IgG antibodies provides passive antibody-mediated immunity to neonates. As most of the IgG transfer occurs during the third trimester, premature infants can have lower IgG and antibody levels compared to term babies, increasing their risk of infection [27].

##### Immunosenescence/inflammageing

Immune system changes that occur with aging may explain, at least in part, the increased risk of infections and disease severity in the elderly population. Immunosenescence typically starts after the age of 50, although it can be seen earlier in chronic inflammatory diseases such as rheumatoid arthritis. The progressive deterioration in immunologic competence leads to weakened antimicrobial immunity, increased susceptibility to infections, impaired vaccine responses, reduced ability to protect against cancers, and possible reactivation of chronic infections such as Shingles [28].

Inflammageing predisposes aging individuals to unopposed tissue inflammation, leading to a number of inflammatory diseases, including atherosclerosis, osteoarthritis, neurodegenerative diseases, and failing wound repair mechanisms [28].

#### Hematologic malignancies

SHG is common in CLL and multiple myeloma (MM). Approximately 25% of CLL patients have SHG at the time of diagnosis, and up to 80% will develop SHG during the course of the disease [1, 2, 4, 29]. Therefore, it is recommended that Ig levels be screened at the time of CLL diagnosis, with periodic, ongoing monitoring of Ig levels thereafter. SHG is seen less frequently in B-cell lymphomas, with reported rates of 7–15% before treatment [2].

Patients with CLL and MM are at increased risk of infection due to SHG as well as other immune defects related to the underlying disease process and BCTT and immunosuppressive agents used for treatment. Multiple aspects of the immune system are defective or aberrant in these patients, and immune changes include neutropenia, mucosal lesions, T-cell dysregulation, and altered complement activation [1, 2, 4, 29].

### Assessment

#### Definitions

When assessing patients with SID, it is helpful to do so within a framework of key definitions that help to standardise evaluations among clinicians.

##### Secondary hypogammaglobulinemia (SHG)

As mentioned earlier, SHG is a humoral SID characterized by reduced Ig levels due to medications or disease processes that lead to decreased antibody production or increased antibody loss. Although various definitions of HG have been used in the literature, the American Academy of Allergy, Asthma, and Immunology (AAAAI) has recently proposed the following definition of HG in adult patients to aid in standardizing future studies: a serum IgG level below < 7 g/L, with further severity stratification into brackets of 0–1.99 g/L, 2.00–3.99 g/L, and 4–6.99 g/L [2]. These stratifications may help to guide clinicians on therapeutic approaches in the future, but to date the published guidelines and recommendations on the diagnosis and management of SHG remain variable. Age-appropriate reference ranges for pediatric patients and variability between laboratories in the reference ranges of Ig should be considered. To better phenotype transient versus persistent SHG, the AAAAI proposes stratification of SHG duration into that lasting 3 to 6 months, 6 to 12 months, 12 to 24 months, and more than 24 months.

##### Relevant infections

Defining ‘‘clinically significant’’ SID has been complicated by a lack of consensus regarding what infection severity constitutes ‘‘severe’’ and what frequency constitutes ‘‘recurrent.’’ To establish a standardized framework for future studies, the AAAAI has proposed the definitions of severe and recurrent infections shown in Table 2 [2]

**Table 2:**
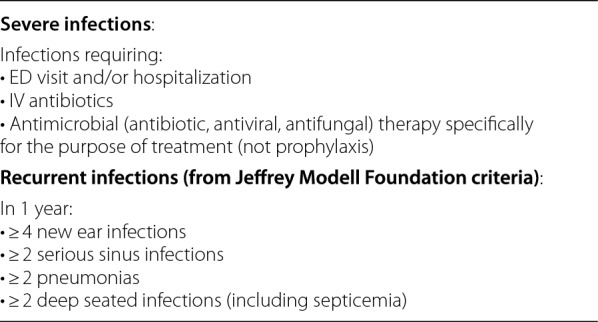
Definitions of severe and recurrent infections

*ED* emergency department, *IV* intravenous

Table adapted from Otani 2022 [2]

#### General review

The initial approach to the assessment of patients with suspected SID should include a thorough history and physical examination to guide immune function testing [2]. The history should focus on potential causes of SID (Fig. 1), infectious complications (including the characteristics, frequency, and severity of infections), and relevant comorbidities such as diabetes mellitus (which can affect neutrophils) or uremia (which affects APCs and phagocytes). During the physical exam, it is also important to pay close attention to skin and mucous membrane health.

#### Investigations specific to T-cell/combined SID

T-cell/combined SID may be suspected in patients presenting with recurrent/severe viral and fungal infections, as well as in those patients who have underlying medical conditions or who receive medications that are known to induce impaired adaptive immune responses. If a T-cell or combined SID is suspected, a complete blood count with differential should be ordered to assess for cytopenias, paying particular attention to lymphocyte counts. T-, B-, and NK-cell (lymphocyte) immunophenotyping may be ordered, which enumerates lymphocyte subsets and identifies which specific cells may be reduced [2].

#### Investigations specific to humoral immunity

Quantitative assessments of Ig levels (IgG, IgA, IgM) are required to evaluate humoral immunity. It is also important to obtain baseline Ig levels at the time of diagnosis of conditions frequently associated with SHG (e.g., CLL and lymphoma), and before and after initiation of medications known to cause SHG (e.g., BCTT, CAR-T-cell therapy) [2]. Serum albumin and total protein levels are useful to identify hypoproteinemia, which raises the suspicion of either malnutrition or protein-losing diseases [2].

Serum protein electrophoresis (SPEP), serum free light chains (FLC), and urine protein electrophoresis (UPEP) can be used to assess for paraproteinemia causing secondary suppression of polyclonal Ig.

More advanced diagnostics, such as pre- and post-vaccine titres to assess the ability to respond to immunization, may also be obtained. The tetanus/diphtheria vaccine can be used to assess protein antigen response, and the pneumococcal vaccine can be used to assess polysaccharide antigen response [2]. The definition of a positive response should be confirmed with the respective laboratories.

Lymphocyte immunophenotyping is also helpful for evaluating immune reconstitution of B-cells after discontinuation of BCTT (i.e., the gradual increase in B-cells over time following treatment discontinuation).

### Management

#### General principles

The management of patients with SID should focus on treating the underlying cause of the immunodeficiency and, where possible, removing the offending agent [1, 2]. Other management principles include measures to reduce exposure to infections (e.g., hand hygiene, avoiding crowded places and/or contact with potentially infections individuals), prophylactic antibiotics, and immunizations. Vaccinations are recommended prior to the initiation of medications known to cause SID and prior to splenectomy. However, live vaccines should be avoided in those with severe immunodeficiency as they can induce vaccine-associated disease. Individuals post splenectomy have an increased susceptibility to invasive and severe infections with encapsulated bacteria (mainly *Streptococcus pneumoniae*). Therefore, vaccinations against *S. pneumoniae, Neisseria meningitidis*, and *Haemophilus influenzae type b* are typically recommended for these patients [1].

#### Immunoglobulin replacement therapy

Immunoglobulin replacement therapy (IGRT) involves the supplementation of normal polyvalent IgG antibodies derived from the plasma of healthy donors. IgG is the primary and most active constituent of all formulations of IGRT, but different products vary in their specific properties. IGRT may be considered for the prevention of infectious complications in patients with SHG [2, 5, 6]. Given the lack of consistent guidelines, initiating IGRT is a complex decision, and shared decision making with the patient and a multidisciplinary care team that includes a clinical immunologist is often required [2]. A paucity of data and limited clinical guidelines also make recommendations for the use of IGRT difficult to generalize for the different causes of SHG. A summary of the AAAAI’s recommendations for select causes of SHG that are based on limited published literature are provided in Table 3 [2]. Table 4 lists some of the IV and SC Ig products carried by Canadian Blood Services [30], and Table 5 lists the Ig products carried by Hema Quebec [31].

**Table 3:**
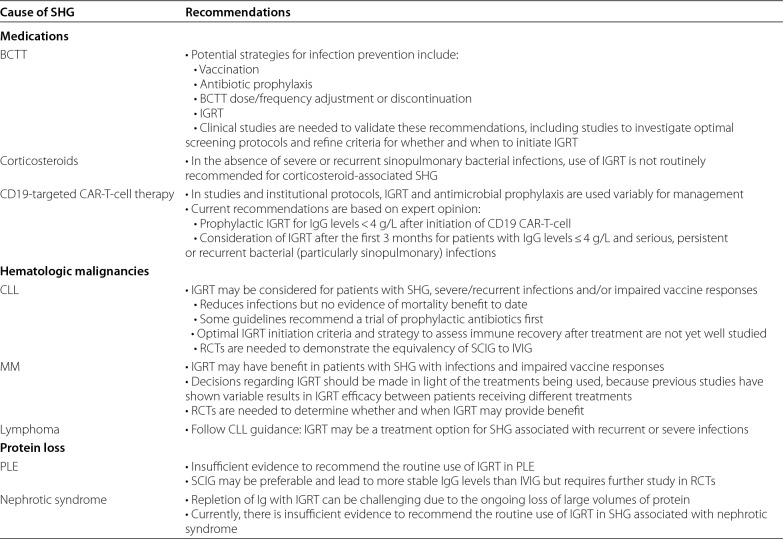
Recommendations for use of IGRT and other infection prevention strategies for specific causes of SHG [2]

BCTT: B-cell targeted therapy; CAR-T-cell: chimeric antigen receptor T-cell; CLL: chronic lymphocytic leukemia; IGRT: immunoglobulin replacement therapy; IVIG: intravenous immunoglobulin; MM: multiple myeloma; PLE: protein-losing enteropathy; RCTs: randomized controlled trials; SCIG: subcutaneous immunoglobulin; SHG: secondary hypogammaglobulinemia

**Table 4:**
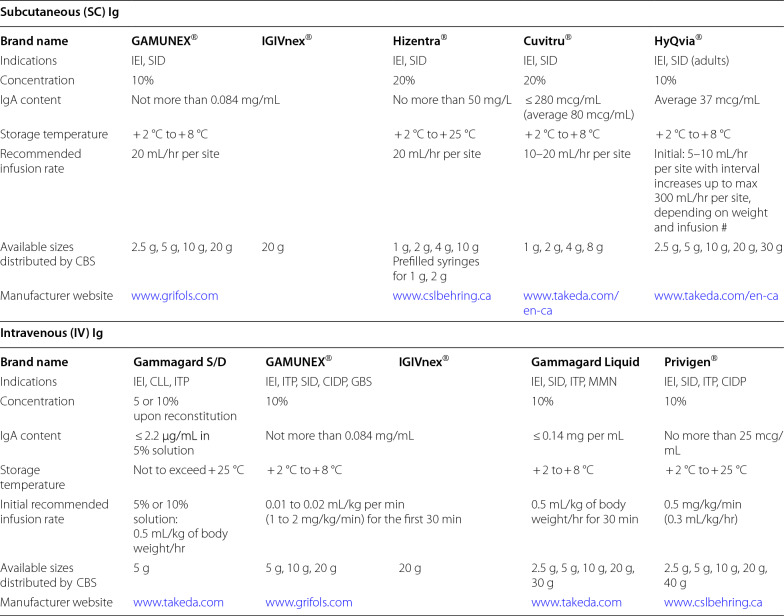
Subcutaneous and Intravenous Ig products carried by Canadian Blood Services [30]

These products may not be available in all cities/provinces across Canada, and other Ig products not listed here may be available. Adapted from Canadian Blood Services 2018. Complete tables available at: https://professionaleducation.blood.ca/en/transfusion/clinical-guide/immune-globulin-products

*IEI* inborn errors of immunity, *SID* secondary immunodeficiency, *ITP* idiopathic thrombocytopenic purpura, *CIDP* chronic inflammatory demyelinating polyneuropathy, *CLL *B-cell chronic lymphocytic leukemia, *GBS* Guillain-Barre Syndrome ; *MMN* multifocal motor neuropathy

**Table 5:**
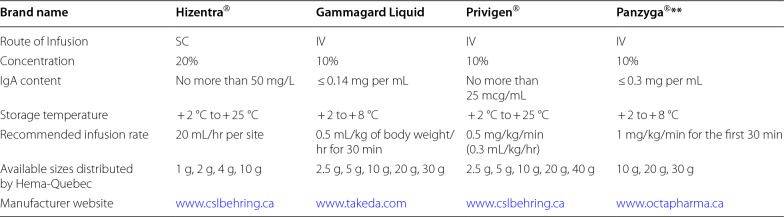
Subcutaneous and Intravenous Ig products carried by Hema-Quebec [31]

These products may not be available in all cities/provinces across Canada, and other Ig products not listed here may be available

**Product will be discontinued once inventory runs out. Adapted from Hema-Quebec (2023). Complete tables available at: https://www.hema-quebec.qc.ca/sang/professionnels-sante/produits-sanguins-stables/index.en.html

IGRT can be administered intravenously (IVIG), typically every 3–4 weeks, or subcutaneously (SCIG). Conventional SCIG is typically administered once to twice weekly, while facilitated SCIG (i.e., SCIG preceded by a hyaluronidase enzyme infusion to increase available subcutaneous space) can be administered every 3–4 weeks. A target trough IgG level of 7–10 g/L is recommended, and IgG should be drawn immediately prior to the next IVIG infusion to assess a “true” trough level. Patients on SCIG have more consistent steady state IgG levels due to their more frequent administration, hence timing of the IgG draw is less well defined.

### Periodic reassessment

For patients receiving prophylactic antibiotics and/or IGRT, periodic reassessments are recommended to determine the ongoing need for therapy and whether a trial of therapy cessation is warranted [2, 5, 6]. During the trial of IGRT cessation, it is important to monitor Ig levels monthly, with periodic clinical reassessment to evaluate whether re-commencement of therapy is required.

### When to refer to a clinical immunologist

SID management can be complex, and clinical guidelines are often limited due to the lack of high-quality data. Additionally, IEI can sometimes be masked by SID. Therefore, a multidisciplinary approach to evaluation and management is recommended for these patients. Referral to a clinical immunologist is recommended for patients with suspected or confirmed SID that may require IGRT or for patients already on IGRT who may benefit from a trial of therapy cessation.

## Conclusions

SID are more common than IEI in adults, and the prevalence of these disorders are increasing annually. The diverse causes of SID and the heterogeneity of the patient populations with these immunodeficiency disorders have presented challenges in conducting high-quality studies to establish best practices for management. Optimal management of patients with SID requires a high index of suspicion for this condition, the appropriate use of diagnostic testing, and a multidisciplinary approach that includes clinical immunology.

## Data Availability

Data sharing not applicable to this article as no datasets were generated or analyzed during the development of this review.

## Abbreviations

AAAAI: American academy of allergy, asthma, and immunology
ALL: Acute lymphoblastic leukemia
APC: Antigen-presenting cell
BCTT: B-cell targeted therapy
CAR-T-cell: Chimeric antigen receptor T-cell
CBS: Canadian blood services
CIDP: Chronic inflammatory demyelinating polyneuropathy
CLL: Chronic lymphocytic leukemia
EBV: Epstein–Barr virus
ED: Emergency department
FL: Follicular lymphoma
FLC: Free light chains
IEI: Inborn errors of immunity
GPA: Granulomatosis with polyangiitis
HG: Hypogammaglobulinemia
HIV: Human immunodeficiency virus
Ig: Immunoglobulin
IgA: Immunoglobulin A
IgM: Immunoglobulin M
IgG: Immunoglobulin G
IGRT: Immunoglobulin replacement therapy
ITP: Idiopathic thrombocytopenic purpura
IV: Intravenous
IVIG: Intravenous immunoglobulin
MM: Multiple myeloma
MMN: Multifocal motor neuropathy
MPA: Microscopic polyangiitis
MS: Multiple sclerosis
NHL: Non-Hodgkin lymphoma
PID: Primary immunodeficiency
PLE: Protein-losing enteropathy
PV: Pemphigus vulgaris
RA: Rheumatoid arthritis
RTX: Rituximab
SARS-CoV-2: Severe acute respiratory syndrome coronavirus 2
SCIG: Subcutaneous immunoglobulin
SHG: Secondary hypogammaglobulinemia
SID: Secondary immunodeficiency
SLE: Systemic lupus erythematosus
SPEP: Serum protein electrophoresis
UPEP: Urine protein electrophoresis
